# Recent Advancements in Poor Graft Function Following Hematopoietic Stem Cell Transplantation

**DOI:** 10.3389/fimmu.2022.911174

**Published:** 2022-06-02

**Authors:** Yan Man, Zhixiang Lu, Xiangmei Yao, Yuemin Gong, Tonghua Yang, Yajie Wang

**Affiliations:** ^1^ Department of Hematology, National Key Clinical Specialty of Hematology, Yunnan Blood Disease Clinical Medical Center, Yunnan Blood Disease Hospital, The First People’s Hospital of Yunnan Province, Kunming, China; ^2^ Department of Hematology, The First Affiliated Hospital of Nanjing Medical University, Jiangsu Province Hospital, Collaborative Innovation Center for Cancer Personalized Medicine, Nanjing, China

**Keywords:** hematopoietic stem cell transplantation, poor graft function, prognosis, bone marrow microenvironment (BMME), hematopoietic stem cell (HSC)

## Abstract

Poor graft function (PGF) is a life-threatening complication that occurs after transplantation and has a poor prognosis. With the rapid development of haploidentical hematopoietic stem cell transplantation, the pathogenesis of PGF has become an important issue. Studies of the pathogenesis of PGF have resulted in some success in CD34^+^-selected stem cell boosting. Mesenchymal stem cells, N-acetyl-l-cysteine, and eltrombopag have also been investigated as therapeutic strategies for PGF. However, predicting and preventing PGF remains challenging. Here, we propose that the seed, soil, and insect theories of aplastic anemia also apply to PGF; CD34^+^ cells are compared to seeds; the bone marrow microenvironment to soil; and virus infection, iron overload, and donor-specific anti-human leukocyte antigen antibodies to insects. From this perspective, we summarize the available information on the common risk factors of PGF, focusing on its potential mechanism. In addition, the safety and efficacy of new strategies for treating PGF are discussed to provide a foundation for preventing and treating this complex clinical problem.

## Introduction

Hematopoietic stem cell transplantation (HSCT) is an effective treatment for malignant hematological diseases. However, delayed or incomplete hematopoietic recovery, also known as poor graft function (PGF), limits the success of HSCT. The definition of PGF is currently controversial. The European Society for Blood and Marrow Transplantation (EBMT) defined PGF as two or three episodes of cytopenia lasting for more than 2 weeks, after day +28 in the presence of donor chimerism > 5% (1). Given the chimerism kinetics and potential for confusion with graft failure (GF), most recent studies based on clinical practice proposed that PGF should be defined as the presence of at least two hematopoietic cell count lines that do not meet the engraftment standard (absolute neutrophil count > 1.5 × 10^9^/L, platelet (PLT) count > 30 × 10^9^/L, hemoglobin > 85g/L) lasting for more than two consecutive weeks beyond day +28 post-HSCT, in the presence of full donor chimerism and primary disease in remission without severe graft-versus-host disease (GVHD) and relapse (2). Secondary poor graft function (sPGF) refers to the loss of donor cells after the initial engraftment. Whereas primary PGF is characterized by no initial donor cell engraftment, it is nearly impossible to recover autologous hematopoiesis; thus, patients with this condition are likely to die of infection and/or other complications and urgently require a secondary transplant.

The cumulative incidence of PGF after allogeneic HSCT (allo-HSCT) varies between 5% and 27% (3–5). Differences in underlying diseases and management strategies affect the incidence of PGF. At Peking University Institute of Hematology, the incidence of primary PGF after unmanipulated haploidentical HSCT (haplo-HSCT) was found to be approximately 5.6% (6, 7), and sPGF developed in 5.7% of patients after allo-HSCT (4). In a prospective study, approximately 15% of patients with severe aplastic anemia (AA) who underwent haplo-SCT developed primary PGF (8). Primary PGF shows a very poor prognosis, with a 1-year overall survival (OS) rate of 25.0% (5) and 2-year OS of 6% in patients without hematopoietic recovery (9). Because of persistent leukocytopenia and thrombocytopenia, PGF is often accompanied by complications such as infection and bleeding, thus increasing the mortality rate. As PGF is a life-threatening complication, new prevention and treatment strategies are urgently needed.

**Figure 1:**
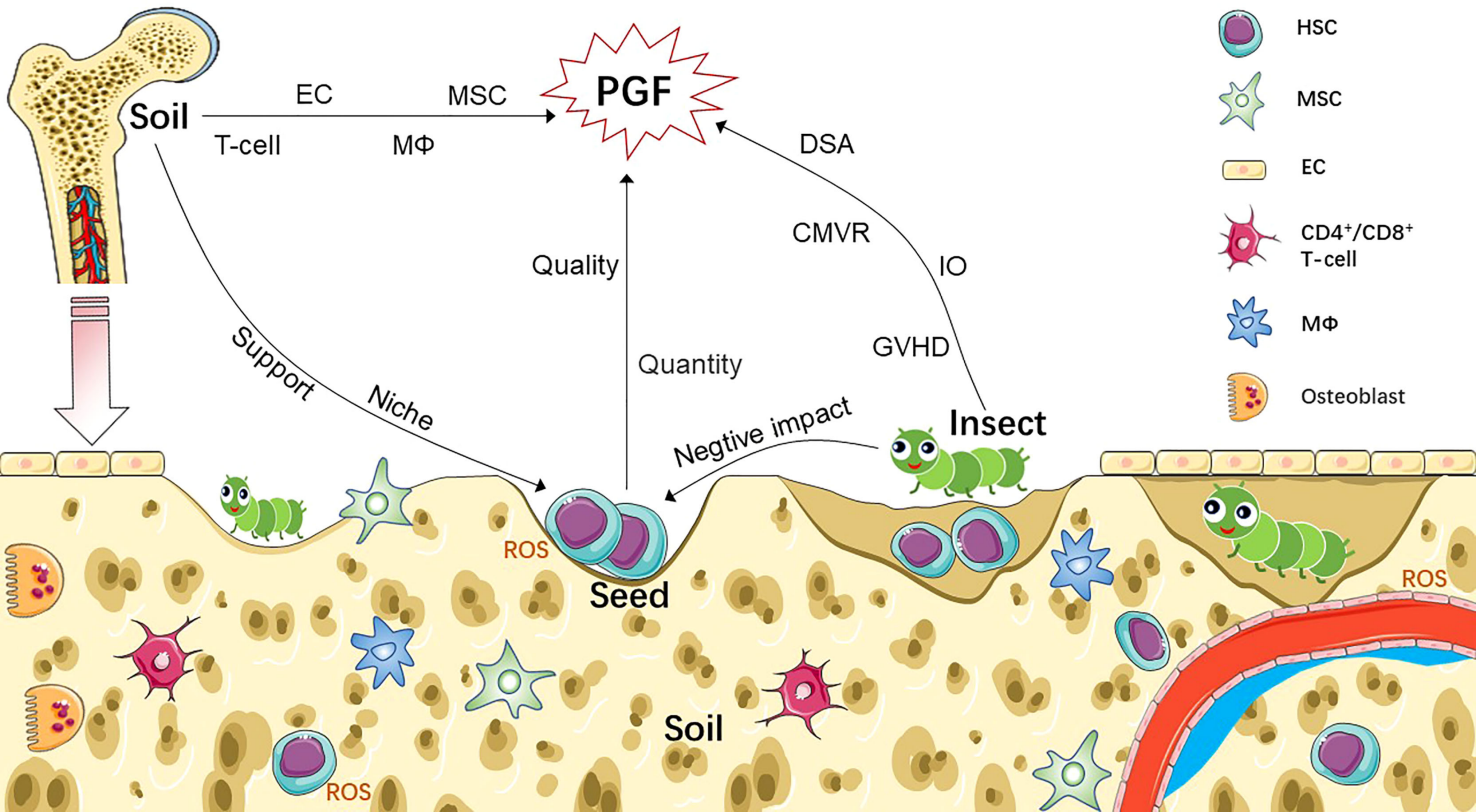
Pathophysiology of PGF based on seed-soil-insect theory. Non-hematopoietic cells (such as MSC, EC) and HSC-derived cells (such as T-cell, macrophages) participate as part of the bone marrow niche, which not only regulate the function of HSC, but also involved in the pathogenesis of PGF after transplantation. Abnormal increased ROS is also contributing to the damage of both HSCs and BM niche of PGF patients. We compare CD34^+^ cells to seeds; bone marrow microenvironment to soil; and various factors associated with immune imbalance to insects, such as GVHD, DSA, CMVR and IO. Seeds, insects and soil interact to form a complex network that leads to PGF. Sufficient number of seeds with good quality and healthy soil are key to successful transplantation. Invading insects damage seeds and soil through knock-on effects, resulting in the occurrence of PGF. CMVR, cytomegalovirus reactivation; DSA, donor-specific anti-human leukocyte antigen antibody; EC, endothelial cell; GVHD, graft-versus-host disease; HSC, hematopoietic stem cell; IO, iron overload; MSC, mesenchymal stem cell; MΦ, macrophage; PGF, poor graft function; ROS, reactive oxygen species.

The occurrence of PGF is related to numerous factors such as primary disease, quality and quantity of hematopoietic stem cells (HSCs), damaged bone marrow (BM) microenvironment, donor-specific anti-human leukocyte antigen (HLA) antibodies (DSA), and viral infection. Based on this information, we hypothesized that the seed, soil, and insect theories of AA pathogenesis can be applied to PGF (**Figure 1**). Based on this theory, we review current research progress on the pathogenesis, prevention, and treatment of PGF.

## The Seed

Similar to seeds, HSCs have the potential of self-renewal and multi-differentiation. Sufficient and functional stem cells have historically been considered key to the success of HSCT. Defects in HSCs, including changes in their quality and quantity, can lead to PGF or GF (**Figure 1**). The donor’s choice directly determines the seed source. Young people are considered better donors than elders, men are superior to women as donors, and matched sibling donors (MSDs) should be prioritized over haploidentical donors (HIDs). However, an older donor/recipient age, female-to-male transplants, and donor-recipient ABO major-mismatch transplants are major risk factors for transplant-related mortality (10). For patients > 50 years old and with high-risk leukemia, HID-HSCT led to a better prognosis compared to MSD-HSCT, with a reduced relapse rate and/or improved leukemia-free survival and OS (11, 12). Interestingly, HID-HSCT may have a stronger graft-versus-leukemia effect than that of MSD-HSCT (12, 13). Thus, in 2021, the Chinese Society of Hematology recommendations noted that “HIDs are the preferred donor choice over MSDs for patients with high-risk leukemia or elderly patients with young offspring donors in experienced centers” (14). The peripheral blood (PB) and BM are the predominant sources of stem cells. There is evidence that in haplo-HSCT with post-transplantation cyclophosphamide using a PB graft, compared with a BM graft, increased the risk of acute GVHD (aGVHD), whereas the 2-year OS, chronic GVHD, relapse, or non-relapse mortality (NRM) were comparable (15, 16). However, another study showed that patients receiving BM had significantly higher 2-year relapse rates compared to those in the PB cohort (36% vs. 16%) (17). Before 2019, mixed grafts of BM + PB were preferred over PB alone in HID-HSCT, because they achieved longer disease-free survival (18). A recent study reported that mixed grafts or PB alone did not influence clinical outcomes (19). Except for haploidentical grafts, rapidly accessible cord blood is a suitable alternative for pediatric patients without HLA-matched donors. Interestingly, the lower incidence of GVHD was not correlated with an increase in long-term survival. For example, in T-cell depleted PB stem cell transplantation, because of the delayed recovery of immune function, the incidence and severity of GVHD are low but are accompanied by an increased risk of infection, relapse, PGF, and transplantation-related mortality, particularly following transplantation with purified CD34^+^ cells (20, 21). As a result, different transplant centers have attempted to use transplant protocols without T-cell depleted conditions, suggesting that unmanipulated transplants are an alternative strategy under haploidentical settings (22). Additionally, a study at Peking University showed that haplo-HSCT without T-cell depleted transplantation has a comparable prognosis as MSD-HSCT (23, 24). Currently, the impact of different graft sources on the incidence of PGF is not well-understood.

A high CD34^+^ cell count is favorable for rapid hematopoietic recovery (8). Cell thresholds were devised to guide whether to carry out further apheresis collection. Currently, most centers use 2 × 10^6^/kg CD34^+^ cells as the minimum threshold. CD34^+^ cells ≥ 5 × 10^6^/kg are currently recommended as the optimal dose by the EBMT. Granulocyte colony-stimulating factor, alone or in combination with chemotherapy, is a standard mobilization regimen for collecting larger amounts of CD34^+^ cells. Plerixafor, a recently approved mobilization agent, is a small-molecule antagonist of CXC chemokine receptor 4 that can effectively block the binding between this chemokine and stromal cell-derived factor-1α, and then release HSCs from the BM to the PB. The combination of granulocyte colony-stimulating factor and plerixafor significantly increased the yield of CD34^+^ cells without causing adverse reactions (25). CD34 molecules were first identified on the surfaces of human hematopoietic stem and progenitor cells (HSPCs). CD34 is also an established marker of other non-hematopoietic cells, including vascular endothelial progenitor cells (EPCs), mesenchymal stem cells (MSCs), and embryonic fibroblasts (26). Thus, the collected “CD34^+^ cells” should not be confused with “CD34^+^ HSPCs.” Introducing new HSPCs markers, such as the signaling lymphocytic activation molecule family (including CD150, CD48, and CD244) (27), may enrich the purer stem cell population. In a nested case-control study of 830 patients, CD34^+^ cell dose < 5 × 10^6^/kg was an independent risk factor for primary PGF (5). A low CD34^+^ cell dose (<median, 2.64 × 10^6^/kg) was also an independent risk factor for sPGF (4). In recent decades, the use of CD34^+^-selected stem cells boosted without preconditioning has significantly improved the prognosis of patients with PGF. In a small-scale study (28), most patients with PGF who received selected CD34^+^ PB stem cells from matched unrelated or mismatched related donors achieved rapid engraftment; more importantly, the procedure was safe, with a low risk of *de novo* grade I–III aGVHD (6%), which was resolved completely. In a long-term follow-up study, CD34^+^-selected infusion without conditioning was feasible in recipients with full donor chimerism and in those with mixed chimerism, whose recovery was similar; patients showing complete recovery had a longer 5-year OS than those with partial recovery (74.4% vs 16.7%) (29). Active infection was considered as the strongest predictor of the efficacy of CD34^+^-selective infusion (29), possibly because of the impaired immune microenvironment caused by inflammation. Cryopreserved products are viable alternatives when additional fresh stem cells cannot be collected. Although the median selection of CD34^+^ counts per kilogram of recipient weight was relatively low (1 × 10^6^/kg), this method achieved promising results; five of the eight cryopreserved product recipients (63%) exhibited a complete hematologic response (25).

It is essential to maintain high-quality stem cells at each step of the transplantation process. An increasing number of studies have been performed to optimize the cell handling, freezing, and thawing steps to ensure stem cell quality (30). Although methods for improving the viability and recovery rate of thawed stem cells are continuously being developed, the procedures still have a negative effect on the product quality and potency (31). Colony assays are the gold standard for stem cell proliferation and differentiation potency *in vitro*, which are used as an additional quality criterion. Watts et al. (32) demonstrated that if the granulocyte-macrophage colony-forming cell dose exceeded 2 × 10^5^/kg after 14 days in culture, total CD34^+^ cells between 1 and 1.9 × 10^6^/kg were also acceptable. When colony assays cannot be performed, cell doses below the threshold should be declared as inadequate, and remobilization and recollection are necessary. However, few centers routinely perform clonal analysis, as these tests require technical support, laboratory standardization, and higher expenses. A rapid method was recently developed to assess the cord blood unit potency for frozen cord blood based on aldehyde dehydrogenase (33). Further studies are necessary to confirm whether this method can be used as an alternative to clonal analysis. In addition, the detection of blood disease-related mutations in donor stem cells prior to transplantation may be of some significance in ensuring the quality of stem cells.

Accumulating evidence has suggested that excess levels of oxygen species (ROS) are responsible for defective hematopoiesis of HSC in patients with PGF, which may be related to disruptions in the stem cell cycle caused by elevated ROS. After transplantation, transiently elevated oxygen tension is beneficial for the rapid proliferation of engrafted HSCs, because it promotes the regeneration of hematopoiesis during the “engraftment window” in the BM niche (34). The BM microenvironment gradually returns to conditions of hypoxic homeostasis with increased oxygen consumption after hematopoietic reconstitution. *In vivo* imaging showed that ROS mediated the initial homing and proliferation of HSCs in lethally irradiated mice, but is not indispensable for long-term hematopoietic reconstitution after transplantation (35). More importantly, Cheng et al. (36, 37) found that in transplanted human HSCs, radiation-induced bystander effects increased ROS levels, contributing to HSC damage and a decrease in transplantation efficiency. It has been hypothesized that forkhead homeobox type O transcription factors are key mediators of ROS regulation in HSCs, contributing to stem cell maintenance and the DNA damage repair response (38). As a negative regulator of forkhead homeobox type O transcription factors, the phosphoinositide 3-kinase (PI3K)/AKT pathway is suppressed in HSCs but activated in hematopoietic progenitors. Activated PI3K/AKT signaling induced HSCs re-entry into the cell cycle, and eventually exhaust HSCs through deletion of phosphatase and tensin homologs (39). In BM, elevated ROS levels induce DNA strand breaks and apoptosis, contributing to the exhaustion of CD34^+^ cells through the p53-p21 pathway in patients with PGF following allo-HSCT, even if the CD34^+^ cells are functionally normal before transplantation (40). Thus, activated p53 can induce HSC depletion. However, Hainaut et al. (41) demonstrated that p53 can also function against ROS-induced DNA damage through its intrinsic redox dependence. Therefore, p53 as a regulator of ROS, playing a dual role in stem cell maintenance.

## The Soil

The BM microenvironment, as the niche for HSC survival, consists of blood vessels, nerves, and a variety of cells that form a complex and precise network to regulate the functional characteristics of HSCs; thus, we compared this microenvironment to soil. In recent years, the mechanisms of various cell and molecular interactions in the BM microenvironment involved in the pathogenesis of PGF have been determined. Huang et al. (42) observed that patients in the sPGF group had marked marrow hypoplasia, and the proportion of CD34^+^ cells, EPCs, CD146^+^ perivascular cells, and endosteal cells were significantly lower than those in the good graft function and healthy control groups. Three years later, they demonstrated that the BM microenvironment was equally damaged in both early and late PGF (43). Recently, a series of translational studies demonstrated that defective autophagy regulated by Beclin-1 (44) or abnormal glycolysis induced by PFKFB3 (45) results in damage to BM endothelial cells (ECs), particularly their decreased hematopoiesis-supporting ability, which is involved in the pathogenesis of PGF post-HSCT. Thus, some transplant events, which may trigger an abnormal increase in ROS in the BM microenvironment, may be essential factors contributing to damage to the BM niche in patients with PGF. As described above, a dysfunctional BM microenvironment may contribute to PGF pathogenesis (**Figure 1**).

### Endothelial Cells

ECs play a crucial role in regulating hematopoiesis by secreting stem cell factor and chemokine ligand 12 in the BM microenvironment (46). An unexpected finding regarding the origin of BM ECs was reported by Plein et al. (47) during early embryogenesis, ECs arise from erythro-myeloid progenitors. Thus, HSCs may provide survival and proliferation signals for EPCs. Accumulating evidence has shown that decreased and dysfunctional BM ECs post-HSCT contribute to the development of PGF (43, 48). Huang et al. (49) provided further evidence that pre-HSCT, BM ECs dysfunction was responsible for the pathogenesis of PGF after haplo-HSCT. BM ECs <0.1% pre-HSCT was an independent risk factor for PGF. Defective hematopoiesis caused by damaged BM ECs is positively correlated with ROS levels (49). Radiation therapy is commonly used in anticancer treatment and myeloablative conditioning regimens before HSCT. Irradiation also severely damages the BM vascular system, particularly sinusoidal ECs, leading to elevated ROS in the BM (35). Notably, elevated ROS levels are observed in ECs and recovering bones in the non-hematopoietic state, even at 2 weeks after sub-lethal irradiation (7 Gy) (50). Huang et al. (48) also showed that the intracellular ROS levels of BM EPCs were elevated after transplantation, and these cells had decreased proliferation and migration capacities. Vasculature reconstruction-mediated hematopoietic engraftment after radiotherapy depends on the expression of vascular cell adhesion molecule 1 on ECs (35), vascular endothelial growth factor receptor 2 signaling in apelin^+^ ECs (51), and vascular endothelial growth factor A provided by transplanted HSPCs (52).

Allogeneic EPCs infusions induced hematopoietic and immune reconstitution in mice, accelerated BM microvascular recovery, and ameliorated GVHD (53, 54). Few studies have reported the infusion of EPCs in humans for clinical treatment, likely because of the limited number of circulating EPCs. In contrast, atorvastatin, a lipid-lowering drug widely used in clinics, was reported to quantitatively improve the impaired function of BM EPCs *in vivo* by downregulating the p38 MAPK pathway in subjects with PGF (48). The antioxidant N-acetyl-l-cysteine can reduce ROS levels both *in vitro* and *in vivo* (49, 55). Prophylactic intervention with oral N-acetyl-l-cysteine not only prevents the occurrence of PGF post-HSCT, but also promotes hematopoietic reconstitution effectively by repairing impaired BM ECs in patients with PGF (49). These results indicate that it is valuable to use antioxidant drugs to improve PGF caused by elevated ROS levels.

### Mesenchymal Stem Cells

MSCs are a type of BM stromal cells with multi-directional differentiation potential, immunoregulatory and hematopoietic support capabilities (56). MSCs show potential for use in treating PGF after HSCT. The mechanisms of MSCs in the pathogenesis of PGF are only beginning to be understood. Compared to in patients with good graft function, BM MSCs from patients with PGF exhibited increased intracellular ROS, higher levels of apoptosis and senescence, and a significantly reduced hematopoiesis-supporting ability *in vitro* (57). Animal experiments and early-phase clinical trials showed that co-infusion of MSCs and HSCs promoted HSC engraftment and improved PGF (58–60). Nevertheless, whether MSCs with immune-suppressive properties increase the incidence of infection and relapse remains controversial. As early as 2007, a clinical study demonstrated that all patients who underwent co-transplantation of *ex vivo* expanded MSC with HLA-disparate CD34^+^ cells showed continuous hematopoietic engraftment, without additional infection compared to in the control group (61). Recently, a systematic review and meta-analysis of children and young individuals showed that MSC co-infusion improved the absolute neutrophil count and PLT engraftment, and greatly reduced the risk of chronic GVHD but had a minimal impact on aGVHD and NRM (59). Similarly, in a systematic review and meta-analysis of haplo-HSCT for severe AA, there was no obvious difference in the 2-year OS, incidence of GVHD and cytomegalovirus (CMV) infection between the MSC co-transplantation group and group not transplanted with MSCs (62). Although the effect of MSCs on PGF was not discussed in the article, co-administration of MSCs with HSCs may not be suitable for patients with severe AA undergoing haplo-HSCT. When infusion of MSCs was performed after transplantation, 17 of 20 patients with primary or secondary PGF experienced hematopoietic recovery when the MSCs were from a third-party donor (60). Moreover, some patients also developed varying degrees of CMV or Epstein-Barr virus infection, acute or chronic GVHD of varying degrees, and even relapse or non-relapse death (60). As summarized above, MSC infusion either before or after transplantation is an effective option for improving PGF, possibly because of their hematopoietic support capabilities. Possible risk factors such as long-term treatment with immunoinhibitors and HLA mismatch may affect the susceptibility to infection. Further studies are needed to determine whether MSCs increase the incidence of infection.

### The Insects

Immune-mediated destruction of hematopoiesis is well-established in the pathogenesis of AA, most likely in the form of AA, an immune imbalance in abnormal hematopoiesis post-HSCT may be responsible for PGF. We compare various factors potentially associated with immune dysregulation post-transplantation, such as GVHD and CMV infection, to insects. GVHD is a fatal complication of allo-HSCT and occurs when donor immunoreactive cells recognize and attack recipient tissue. Grade III–IV GVHD is significantly associated with PGF development (9). Various factors that lead to GVHD, such as ongoing immune stimulation, may prevent hematopoietic reconstitution and exhaust hematopoietic precursor cells, eventually resulting in PGF development after transplantation.

Both CD4^+^ and CD8^+^ T cells are dramatically polarized towards the type 1 immune response in patients with PGF after allo-HSCT (63, 64). Thus, dysregulated T cell responses in the BM immune microenvironment may be involved in the pathogenesis of PGF after HSCT. Luo et al. (65) found that M2 macrophages (MΦs) supported and M1 MФ suppressed HSC self-renewal and expansion *in vitro*. MΦs derived from patients with PGF exhibited significantly increased M1 and decreased M2 relative to those from patients with good graft function and healthy donors (66). Furthermore, the function of MΦs was impaired, characterized by reduced hematopoiesis-supporting ability, resulting in BM CD34^+^ cell dysfunction through p38 MAPK pathway upregulation, and aggravated pancytopenia in patients with PGF (66). Moreover, Zhao et al. (67) confirmed the opposing effects of M1 and M2 MФs on megakaryocytes: M1 MФs inhibit whereas M2 MФs promote MK maturation and platelet formation. MФs in patients with prolonged isolated thrombocytopenia also polarized towards M1, and unbalanced MΦs polarization impaired the megakaryopoiesis-supporting ability of BM MФs, which was rescued by activation of the PI3K-AKT pathway. Further studies are needed to determine how these dysfunctional immune cells interact with other cellular elements or directly affect hematopoiesis, which may provide insight into the underlying molecular mechanisms and potential therapeutic strategies for patients with PGF after HSCT.

Thrombocytopenia caused by ongoing immune attacks limits the recovery of PGF. The thrombopoietin (TPO) receptor agonist (TPO-RA), romiplostim and eltrombopag showed promising results for treating immune thrombocytopenia (ITP). Additionally, the combination of eltrombopag with standard immunosuppressive therapy (horse antithymocyte globulin plus cyclosporine) shows great potential for treating severe AA (68), because it improves the rate, rapidity, and strength of hematologic responses in severe AA without causing toxic effects. Moreover, eltrombopag has been successfully used to treat PFG after allo-HSCT (69). Eltrombopag has changed the paradigm of AA treatment, however, some patients do not respond to this treatment. Nakao et al. (70) reported that high-dose romiplostim (20 μg/kg) was highly effective in patients with AA refractory to eltrombopag. Avatrombopag is a second-generation TPO-RA approved for second-line treatment of primary chronic ITP. A multicenter study performed in the United States confirmed that: patients with ITP who previously used other TPO-RAs (eltrombopag or romiplostim) and responded poorly exhibited a high response rate to avatrombopag (71). Thus, TPO-RA may be another option for treating PGF.

### Donor-Specific Anti-HLA Antibodies

Antibody-mediated graft rejection is considered to cause GF. Various preformed antibodies are detectable in patients after allo-HSCT (72). DSA refers to specific antibodies corresponding to a mismatched antigen produced in patients after organ/tissue transplantation. Circulating DSA can lead to hyperacute rejection and thus is an important factor affecting HSC engraftment and is related to PGF, particularly primary PGF (7, 73). In a retrospective analysis of 394 patients who underwent haplo-HSCT, DSA with median fluorescence intensity (MFI) ≥1000 was significantly correlated with prolonged isolated thrombocytopenia (hazard ratio 3.262; *P* = 0.009) (74). DSA with MFI ≥ 1000 was also considered associated with the cumulative incidence of neutrophil engraftment for 60 days after single-unit cord blood transplantation (*P* = 0.03) (75). Ciurea et al. (76) found that patients with high DSA levels (> 5000 MFI) and complement-binding DSA antibodies (C1q-positive) exhibited graft rejection at the time of transplantation, whereas patients whose C1q became negative after desensitization therapy were successfully engrafted by donor cells. In the absence of effective salvage approaches, the mortality rate of patients with GF is close to 100%, particularly after haplo-HSCT. A second early transplant can often successfully salvage GF (77, 78). The absence of DSA was associated with lower NRM and improved OS (79). These studies support the consensus guidelines from the EBMT, suggesting that DSA and C1q levels must be monitored to further assess the risk of allograft in patients with DSAs ≥ 1,000 MFI (80).

Reducing DSA levels is essential for preventing primary PGF. Plasma exchange, rituximab, PLT transfusions, bortezomib, and immunoglobulin are often used clinically to decrease DSA levels. The rate of granulocyte reconstruction in the DSA-positive group was lower than that in the DSA-negative group after desensitization therapy (81–83). During HLA-mismatched HSCT, a single dose of rituximab was effective for desensitization and prevented the onset of primary PGF in DSA-positive patients, whereas bortezomib and immunoglobulin alone showed a limited ability to rapidly decrease DSA levels (81). Patients were desensitized to a DSA level < 2000 MFI after combination therapy with rituximab and/or plasmapheresis (83). However, DSA may rapidly rebound at any time. In a case report by Hassan et al. (84), during hematopoietic progenitor cell transplantation, DSA unexpectedly rebounded and rapidly increased during desensitization with repeated plasma exchange and immunoglobulin, finally leading to primary PGF. However, the cause of this phenomenon remains unclear. As an IgG-degrading enzyme of *Streptococcus pyogenes*, imlifidase can inhibit complement-and FcγR-mediated effector functions by cleaving donor-specific IgG into Fc and F (ab’) 2 fragments (85). Endoglycosidase of *S. pyogenes* (EndoS) reduces the affinity of IgG for FcγRs by specifically hydrolyzing glycans of all subclasses of human IgG (86). Both imlifidase and EndoS partially block DSA’s function. To further reduce the titer and inhibit the effector functions of residual DSA, Anderson et al. (87) demonstrated that a combination of imlifidase and EndoS can be used to inactivate DSA and inhibit DSA-mediated killing of donor BM cells in allogeneic BM transplantation. Further studies are needed to confirm whether enzyme-mediated DSA blocking prevents antibody rebound.

### Cytomegalovirus Infection

CMV and Epstein-Barr virus reactivation are independent risk factors for sPGF within the first 100 days of allo-HSCT (4, 88). CMV and Epstein-Barr virus co-reactivation not only leads to a shorter 1-year OS and leukemia-free survival, but also results in poor regulatory T cell reconstitution at day 30 after allo-HSCT (89). CMV reactivation (CMVR) after HSCT can lead to a variety of common life-threatening infectious complications such as pneumonia, retinitis, or sPGF. The prognosis of CMVR and immune reconstitution of CMV-specific T-cells are closely related (90, 91). In the first year post-transplantation, clonal expansion of CMV-specific effector memory T-cells drives the reconstitution of CD4^+^ and CD8^+^ T-cells. Furthermore, the heterogeneity and diversity of the remaining T-cell repertoire are impaired in patients who experience reactivation (92, 93).

Previous studies suggested that CMVR after transplantation is strongly associated with aGVHD. Recent evidence demonstrated that mismatches in major or minor histocompatibility antigens promote CMV disease by inducing non-cognate transplantation tolerance, which inhibits the efficient reconstitution of antiviral CD8^+^ T cells, eventually resulting in cytopathogenic tissue infections (90). CMVR was associated with an increased risk of NRM with or without GVHD; however, the interaction between GVHD and CMVR was not significant (*P* = 0.326) (94). Therefore, aGVHD does not appear to be necessary for CMVR. However, the results of different studies varied based on the baseline characteristics. Single-center studies reported that CMVR after allo-HSCT is positively correlated with a decreased risk of relapse in acute myelocytic leukemia but not in other hematological malignancies. This benefit is of little significance considering the increased NMR and overall mortality (95). Another study of the Center for International Blood and Marrow Transplant Research database confirmed CMVR as a risk factor for poor prognosis, but showed no benefit of CMVR on the relapse of hematologic disease (96). CMV peak titers, disease stage, and T-cell depletion with antithymocyte globulin, which are associated with immunity, may modulate the impact of CMVR on leukemia relapse (97, 98).

In the era of PCR-based monitoring, universal prophylaxis or preemptive therapy strategy is typically adopted to prevent and treat CMV infections after HSCT (99). In the 1980s and 1990s, high-dose acyclovir and valacyclovir showed limited efficacy in preventing CMV disease (100). Some agents have been used for decades to control CMV infection and lead to significant toxicity. For example, ganciclovir (101, 102) is hemotoxic and frequently leads to secondary bacterial and fungal infections. Additionally, foscarnet (103) and cidofovir (102) exhibit severe renal toxicity. The introduction of letermovir (LMV) is an important advancement. As a CMV DNA terminase complex inhibitor, LMV can be administered orally and intravenously and has no myelotoxicity or nephrotoxicity (102, 104). Co-administration of cyclosporine increased the bioavailability of LMV from 35% to 85%; thus, lower doses are required in patients taking cyclosporine to prevent GVHD. Increasing evidence has shown that prophylactic LMV treatment effectively prevents the development of refractory or resistant CMV infections and ultimately decreases transplant-related mortality (105–107). In addition, Zamora et al. (108) provided initial evidence that compared with ganciclovir preemption, LMV prophylaxis-associated CMV antigen exposure reduction delays CMV-specific T-cell reconstitution after HSCT.

Virological monitoring of CMV in the blood plasma is routinely performed using quantitative PCR, but there is currently no consensus on the plasma viral load threshold when initiating CMV preemptive treatment. Real-time CMV-specific cell-mediated immunity responses were successfully applied to predict clinical CMV events and guide the early discontinuation of antivirals (109). Future strategies may involve vaccination dependent on functional reconstitution of CD4^+^ T cells and B cells (91, 110), other novel antiviral agents [maribavir (111), CMX001 (112)], antibodies that block cell-to-cell spread and kill latently infected cells (113, 114), and adoptive cell therapy not limited by GVHD and steroids (115).

### Iron Overload

Iron is a raw material for hematopoiesis. Long-term blood transfusion and inflammation are the most common factors leading to iron overload (IO) in patients with hematological malignancies. Studies have shown mixed results regarding the impact of IO pre-transplant on PGF and prognosis, possibly because of differences in marker selection and baseline data of the study population. Serum ferritin (SF) is a biomarker of IO. In two prospective studies by Zhao et al. (5) and Malki et al. (116), SF > 2000 ng/mL before HSCT was identified as an independent risk factor for primary PGF and a strong poor prognostic factor. In a subsequent prospective multicenter study, patients with SF > 1500 ng/mL before the start of conditioning with allo-HSCT had an inferior OS (hazard ratio, 2.5, CI = 1.5-4.1, *P* = 0.0005) and progression-free survival (hazard ratio, 2.4, CI = 1.6-3.8, *P* < 0.0001) (117). In contrast, in a prospective cohort study using liver magnetic resonance imaging to quantify the liver iron content, there was no significant correlation between IO (liver iron content >1.8 mg/g) before allo-HSCT and the cumulative incidence of multiple complications, OS, or NRM after HSCT (118). Interestingly, using SF or the liver iron content as a marker of IO revealed that IO was not related to the occurrence of acute or chronic GVHD (117, 118). Hepcidin expressed by the liver, it modulates iron absorption and release and is overexpressed when IO decreases these processes, and the erythropoiesis demands can eventually not be met (119). The rates of OS and PLT engraftment were significantly lower in the high hepcidin group than in the low hepcidin group (120). Hepcidin may be an alternative marker of IO to predict delayed PLT engraftment after allo-HSCT; however, there is currently no accepted validated method for evaluating hepcidin. Growth differentiation factor 15 belongs to the transforming growth factor-beta superfamily and has been proposed as an erythroid regulator involved in hepcidin suppression (121). Erythroferrone is a new erythroid regulator of hepcidin produced by erythroid precursors in response to stress erythropoiesis *via* the Jak2/Stat5 signaling pathway (122).

Zhao et al. (123) reported that IO damaged the erythroid colony-forming capacity of normal HSPCs and reduced the frequency of abnormal HSPCs in MDS mice. Impaired erythroid HSPCs are, at least in part, related to growth differentiation factor 15-induced ROS (123). In addition, IO contributes to MSC damage through the AMPK/MFF/Drp1 pathway, which displays increased cell apoptosis, decreased cell viability, and extensive autophagy, all of which are ROS-dependent (124). Excess iron levels can compromise BM stromal cells, inhibit erythropoietin and thrombopoietin levels, and disrupt hematopoietic function by increasing oxidative stress (125). Thus, the holistic situation during treatment and the link between IO and ROS should be considered. Currently, deferiprone and deferasirox are the most commonly used iron-chelating agents for removing IO in the clinic (126). Eltrombopag is also a powerful iron chelator with intracellular iron mobilization characteristics that can reduce iron-induced ROS and stimulate stem cell hematopoiesis independently of the TPO receptor (127, 128). Tang et al. (69) preliminarily verified the feasibility of treating sPGF post-allo-HSCT with eltrombopag in a retrospective analysis. Co-administration of eltrombopag with clinically available chelators, such as deferasirox, may be an effective means for indirect PGF treatment (127). Additionally, upregulation of ferritin (129) and transferrin infusion (130) improve BM hematopoietic function induced by IO in mice; further studies are required to confirm its clinical feasibility.

## Conclusion

The pathogenesis of PGF involves a complex, interlocking network. Future approaches to address PGF should focus on optimizing seeds, improving soil, and killing insects, emphasizing the importance of early detection and treatment to avoid PGF. With regard to seeds, a certain scale of research has focused on improving the quantity; and quality inspection before infusion should be performed to prevent the occurrence of PGF. Emerging research attempts to describe the relationship between the BM microenvironment and PGF, and improve the understanding of how various stromal cells, related factors, and abnormally activated transduction pathways interact to promote the initiation and development of PGF, these may lead to the development of prevention and treatment strategies. From a superficial perspective, different “insects” have different impacts on PGF through different mechanisms. These insects are products of immunodeficiencies. Therefore, new drugs that kill insects and focus on targeted immune modulation are needed. The quality of donor CD34^+^ cells should be routinely evaluated prior to transplantation. If possible, to predict the incidence of PGF, the content and functional status of donor cells and level of oxidative stress in the recipient’s BM microenvironment should be detected to determine whether to continue transplantation or administer corresponding treatment in advance. These steps may be feasible preventive measures for PGF and require further validation.

## Author Contributions

YW and TY conceptualized the outline and topic of the article. YM, XY, and ZL participated in collecting literature and draft manuscripts. ZL designed the figure. YM and XY analyzed the literature and made the figure. YW, TY, and YG coordinated the revision. All authors contributed to the article and approved the submitted version.

## Funding

This work was supported by the National Natural Science Foundation of China (82070173, 82060810,81900109), the Basic Applied Research Projects of Yunnan Province (202101AW070017), the Doctoral Research Fund of the First People’s Hospital of Yunnan Province (KHBS-2020-007), the Open Project of Yunnan Blood Disease Clinical Medical Center (2020LCZXKF-XY14).

## Conflict of Interest

The authors declare that the research was conducted in the absence of any commercial or financial relationships that could be construed as a potential conflict of interest.

## Publisher’s Note

All claims expressed in this article are solely those of the authors and do not necessarily represent those of their affiliated organizations, or those of the publisher, the editors and the reviewers. Any product that may be evaluated in this article, or claim that may be made by its manufacturer, is not guaranteed or endorsed by the publisher.

## References

[1] Carreras E Dufour C Mohty M Kröger N. The Ebmt Handbook: Hematopoietic Stem Cell Transplantation and Cellular Therapies. In: Carreras E Dufour C Mohty M Kroger N, editors. The Ebmt Handbook: Hematopoietic Stem Cell Transplantation and Cellular Therapies. Cham (CH: Springer) (2019). 32091673

[2] McLornan DP Hernandez-Boluda JC Czerw T Cross N Joachim Deeg H Ditschkowski M. Allogeneic Haematopoietic Cell Transplantation for Myelofibrosis: Proposed Definitions and Management Strategies for Graft Failure, Poor Graft Function and Relapse: Best Practice Recommendations of the Ebmt Chronic Malignancies Working Party. Leukemia (2021) 35(9):2445–59. doi: 10.1038/s41375-021-01294-2 34040148

[3] Alchalby H Yunus DR Zabelina T Ayuk F Kroger N. Incidence and Risk Factors of Poor Graft Function After Allogeneic Stem Cell Transplantation for Myelofibrosis. Bone Marrow Transplant (2016) 51(9):1223–7. doi: 10.1038/bmt.2016.98 27088376

[4] Sun YQ Wang Y Zhang XH Xu LP Liu KY Yan CH. Virus Reactivation and Low Dose of Cd34+ Cell, Rather Than Haploidentical Transplantation, Were Associated With Secondary Poor Graft Function Within the First 100 Days After Allogeneic Stem Cell Transplantation. Ann Hematol (2019) 98(8):1877–83. doi: 10.1007/s00277-019-03715-w 31144019

[5] Zhao Y Gao F Shi J Luo Y Tan Y Lai X. Incidence, Risk Factors, and Outcomes of Primary Poor Graft Function After Allogeneic Hematopoietic Stem Cell Transplantation. Biol Blood Marrow Transplant (2019) 25(9):1898–907. doi: 10.1016/j.bbmt.2019.05.036 31176790

[6] Sun YQ He GL Chang YJ Xu LP Zhang XH Han W. The Incidence, Risk Factors, and Outcomes of Primary Poor Graft Function After Unmanipulated Haploidentical Stem Cell Transplantation. Ann Hematol (2015) 94(10):1699–705. doi: 10.1007/s00277-015-2440-x 26152553

[7] Chang YJ Zhao XY Xu LP Zhang XH Wang Y Han W. Donor-Specific Anti-Human Leukocyte Antigen Antibodies Were Associated With Primary Graft Failure After Unmanipulated Haploidentical Blood and Marrow Transplantation: A Prospective Study With Randomly Assigned Training and Validation Sets. J Hematol Oncol (2015) 8:84. doi: 10.1186/s13045-015-0182-9 26156584 PMC4496923

[8] Arcuri LJ Nabhan SK Cunha R Nichele S Ribeiro AAF Fernandes JF. Impact of Cd34 Cell Dose and Conditioning Regimen on Outcomes After Haploidentical Donor Hematopoietic Stem Cell Transplantation With Post-Transplantation Cyclophosphamide for Relapsed/Refractory Severe Aplastic Anemia. Biol Blood Marrow Transplant (2020) 26(12):2311–7. doi: 10.1016/j.bbmt.2020.09.007 32949751

[9] Prabahran A Koldej R Chee L Wong E Ritchie D. Evaluation of Risk Factors for and Subsequent Mortality From Poor Graft Function (Pgf) Post Allogeneic Stem Cell Transplantation. Leuk Lymphoma (2021) 62(6):1482–9. doi: 10.1080/10428194.2021.1872072 33522344

[10] Wang Y Wu DP Liu QF Xu LP Liu KY Zhang XH. Donor and Recipient Age, Gender and Abo Incompatibility Regardless of Donor Source: Validated Criteria for Donor Selection for Haematopoietic Transplants. Leukemia (2018) 32(2):492–8. doi: 10.1038/leu.2017.199 28642591

[11] Wang Y Liu QF Wu DP Xu LP Liu KY Zhang XH. Improved Survival After Offspring Donor Transplant Compared With Older Aged-Matched Siblings for Older Leukaemia Patients. Br J Haematol (2020) 189(1):153–61. doi: 10.1111/bjh.16303 31696939

[12] Yu S Huang F Wang Y Xu Y Yang T Fan Z. Haploidentical Transplantation Might Have Superior Graft-Versus-Leukemia Effect Than Hla-Matched Sibling Transplantation for High-Risk Acute Myeloid Leukemia in First Complete Remission: A Prospective Multicentre Cohort Study. Leukemia (2020) 34(5):1433–43. doi: 10.1038/s41375-019-0686-3 31831845

[13] Guo H Chang YJ Hong Y Xu LP Wang Y Zhang XH. Dynamic Immune Profiling Identifies the Stronger Graft-Versus-Leukemia (Gvl) Effects With Haploidentical Allografts Compared to Hla-Matched Stem Cell Transplantation. Cell Mol Immunol (2021) 18(5):1172–85. doi: 10.1038/s41423-020-00597-1 PMC8093297 33408344

[14] Zhang XH Chen J Han MZ Huang H Jiang EL Jiang M. The Consensus From the Chinese Society of Hematology on Indications, Conditioning Regimens and Donor Selection for Allogeneic Hematopoietic Stem Cell Transplantation: 2021 Update. J Hematol Oncol (2021) 14(1):145. doi: 10.1186/s13045-021-01159-2 34526099 PMC8441240

[15] Ruggeri A Labopin M Bacigalupo A Gulbas Z Koc Y Blaise D. Bone Marrow Versus Mobilized Peripheral Blood Stem Cells in Haploidentical Transplants Using Posttransplantation Cyclophosphamide. Cancer (2018) 124(7):1428–37. doi: 10.1002/cncr.31228 29360162

[16] Yu X Liu L Xie Z Dong C Zhao L Zhang J. Bone Marrow Versus Peripheral Blood as a Graft Source for Haploidentical Donor Transplantation in Adults Using Post-Transplant Cyclophosphamide-A Systematic Review and Meta-Analysis. Crit Rev Oncol Hematol (2019) 133:120–8. doi: 10.1016/j.critrevonc.2018.05.017 30661648

[17] Sharma N Faisal MS Zhao Q Jiang J Elder P Benson DM. Outcomes of Bone Marrow Compared to Peripheral Blood for Haploidentical Transplantation. J Clin Med (2021) 10(13):2843. doi: 10.3390/jcm10132843 34199028 PMC8268935

[18] Xu L Chen H Chen J Han M Huang H Lai Y. The Consensus on Indications, Conditioning Regimen, and Donor Selection of Allogeneic Hematopoietic Cell Transplantation for Hematological Diseases in China-Recommendations From the Chinese Society of Hematology. J Hematol Oncol (2018) 11(1):33. doi: 10.1186/s13045-018-0564-x 29495966 PMC5833104

[19] Ma YR Zhang X Xu L Wang Y Yan C Chen H. G-Csf-Primed Peripheral Blood Stem Cell Haploidentical Transplantation Could Achieve Satisfactory Clinical Outcomes for Acute Leukemia Patients in the First Complete Remission: A Registered Study. Front Oncol (2021) 11:631625. doi: 10.3389/fonc.2021.631625 33791217 PMC8005750

[20] Elfeky R Shah RM Unni MNM Ottaviano G Rao K Chiesa R. New Graft Manipulation Strategies Improve the Outcome of Mismatched Stem Cell Transplantation in Children With Primary Immunodeficiencies. J Allergy Clin Immunol (2019) 144(1):280–93. doi: 10.1016/j.jaci.2019.01.030 30731121

[21] Ciurea SO Mulanovich V Saliba RM Bayraktar UD Jiang Y Bassett R. Improved Early Outcomes Using a T Cell Replete Graft Compared With T Cell Depleted Haploidentical Hematopoietic Stem Cell Transplantation. Biol Blood Marrow Transplant (2012) 18(12):1835–44. doi: 10.1016/j.bbmt.2012.07.003 PMC4320643 22796535

[22] Chang YJ Huang XJ. Haploidentical Bone Marrow Transplantation Without T-Cell Depletion. Semin Oncol (2012) 39(6):653–63. doi: 10.1053/j.seminoncol.2012.09.003 23206842

[23] Chen H Liu KY Xu LP Chen YH Han W Zhang XH. Haploidentical Hematopoietic Stem Cell Transplantation Without *in Vitro* T Cell Depletion for the Treatment of Philadelphia Chromosome-Positive Acute Lymphoblastic Leukemia. Biol Blood Marrow Transplant (2015) 21(6):1110–6. doi: 10.1016/j.bbmt.2015.02.009 25698612

[24] Lu DP Dong L Wu T Huang XJ Zhang MJ Han W. Conditioning Including Antithymocyte Globulin Followed by Unmanipulated Hla-Mismatched/Haploidentical Blood and Marrow Transplantation Can Achieve Comparable Outcomes With Hla-Identical Sibling Transplantation. Blood (2006) 107(8):3065–73. doi: 10.1182/blood-2005-05-2146 16380454

[25] Ghobadi A Fiala MA Ramsingh G Gao F Abboud CN Stockerl-Goldstein K. Fresh or Cryopreserved Cd34(+)-Selected Mobilized Peripheral Blood Stem and Progenitor Cells for the Treatment of Poor Graft Function After Allogeneic Hematopoietic Cell Transplantation. Biol Blood Marrow Transplant (2017) 23(7):1072–7. doi: 10.1016/j.bbmt.2017.03.019 PMC5515540 28323004

[26] Sidney LE Branch MJ Dunphy SE Dua HS Hopkinson A. Concise Review: Evidence for Cd34 as a Common Marker for Diverse Progenitors. Stem Cells (2014) 32(6):1380–9. doi: 10.1002/stem.1661 PMC4260088 24497003

[27] Kiel MJ Yilmaz OH Iwashita T Yilmaz OH Terhorst C Morrison SJ. Slam Family Receptors Distinguish Hematopoietic Stem and Progenitor Cells and Reveal Endothelial Niches for Stem Cells. Cell (2005) 121(7):1109–21. doi: 10.1016/j.cell.2005.05.026 15989959

[28] Mainardi C Ebinger M Enkel S Feuchtinger T Teltschik HM Eyrich M. Cd34(+) Selected Stem Cell Boosts Can Improve Poor Graft Function After Paediatric Allogeneic Stem Cell Transplantation. Br J Haematol (2018) 180(1):90–9. doi: 10.1111/bjh.15012 29205259

[29] Cuadrado MM Szydlo RM Watts M Patel N Renshaw H Dorman J. Predictors of Recovery Following Allogeneic Cd34+-Selected Cell Infusion Without Conditioning to Correct Poor Graft Function. Haematologica (2020) 105(11):2639–46. doi: 10.3324/haematol.2019.226340 PMC7604618 33131253

[30] Watts MJ Linch DC. Optimisation and Quality Control of Cell Processing for Autologous Stem Cell Transplantation. Br J Haematol (2016) 175(5):771–83. doi: 10.1111/bjh.14378 27748518

[31] Purtill D Antonenas V Chiappini P Tong D O'Flaherty E Bajel A. Variable Cd34+ Recovery of Cryopreserved Allogeneic Hpc Products: Transplant Implications During the Covid-19 Pandemic. Blood Adv (2020) 4(17):4147–50. doi: 10.1182/bloodadvances.2020002431 PMC7479963 32886750

[32] Watts MJ Ings SJ Balsa C Antonio A Hack S Linch DC. Re-Evaluation of Progenitor Thresholds and Expectations for Haematopoietic Recovery Based on an Analysis of 810 Autologous Transplants: Implications for Quality Assurance. Br J Haematol (2016) 175(4):673–6. doi: 10.1111/bjh.14276 27507229

[33] Shoulars K Noldner P Troy JD Cheatham L Parrish A Page K. Development and Validation of a Rapid, Aldehyde Dehydrogenase Bright-Based Cord Blood Potency Assay. Blood (2016) 127(19):2346–54. doi: 10.1182/blood-2015-08-666990 PMC4865591 26968535

[34] Moirangthem RD Singh S Adsul A Jalnapurkar S Limaye L Kale VP. Hypoxic Niche-Mediated Regeneration of Hematopoiesis in the Engraftment Window Is Dominantly Affected by Oxygen Tension in the Milieu. Stem Cells Dev (2015) 24(20):2423–36. doi: 10.1089/scd.2015.0112 PMC4599134 26107807

[35] Lewandowski D Barroca V Duconge F Bayer J Van Nhieu JT Pestourie C. *In Vivo* Cellular Imaging Pinpoints the Role of Reactive Oxygen Species in the Early Steps of Adult Hematopoietic Reconstitution. Blood (2010) 115(3):443–52. doi: 10.1182/blood-2009-05-222711 19797522

[36] Shen H Yu H Liang PH Cheng H XuFeng R Yuan Y. An Acute Negative Bystander Effect of Gamma-Irradiated Recipients on Transplanted Hematopoietic Stem Cells. Blood (2012) 119(15):3629–37. doi: 10.1182/blood-2011-08-373621 PMC3325047 22374698

[37] Hu L Yin X Zhang Y Pang A Xie X Yang S. Radiation-Induced Bystander Effects Impair Transplanted Human Hematopoietic Stem Cells *Via* Oxidative DNA Damage. Blood (2021) 137(24):3339–50. doi: 10.1182/blood.2020007362 PMC8233686 33881475

[38] Storz P. Forkhead Homeobox Type O Transcription Factors in the Responses to Oxidative Stress. Antioxid Redox Signal (2011) 14(4):593–605. doi: 10.1089/ars.2010.3405 20618067 PMC3038124

[39] Yilmaz OH Valdez R Theisen BK Guo W Ferguson DO Wu H. Pten Dependence Distinguishes Haematopoietic Stem Cells From Leukaemia-Initiating Cells. Nature (2006) 441(7092):475–82. doi: 10.1038/nature04703 16598206

[40] Kong Y Song Y Hu Y Shi MM Wang YT Wang Y. Increased Reactive Oxygen Species and Exhaustion of Quiescent Cd34-Positive Bone Marrow Cells May Contribute to Poor Graft Function After Allotransplants. Oncotarget (2016) 7(21):30892–906. doi: 10.18632/oncotarget.8810 PMC5058726 27105530

[41] Hafsi H Hainaut P. Redox Control and Interplay Between P53 Isoforms: Roles in the Regulation of Basal P53 Levels, Cell Fate, and Senescence. Antioxid Redox Signal (2011) 15(6):1655–67. doi: 10.1089/ars.2010.3771 21194382

[42] Kong Y Chang YJ Wang YZ Chen YH Han W Wang Y. Association of an Impaired Bone Marrow Microenvironment With Secondary Poor Graft Function After Allogeneic Hematopoietic Stem Cell Transplantation. Biol Blood Marrow Transplant (2013) 19(10):1465–73. doi: 10.1016/j.bbmt.2013.07.014 23879970

[43] Kong Y Wang YT Hu Y Han W Chang YJ Zhang XH. The Bone Marrow Microenvironment Is Similarly Impaired in Allogeneic Hematopoietic Stem Cell Transplantation Patients With Early and Late Poor Graft Function. Bone Marrow Transplant (2016) 51(2):249–55. doi: 10.1038/bmt.2015.229 26437066

[44] Lyu ZS Cao XN Wen Q Mo XD Zhao HY Chen YH. Autophagy in Endothelial Cells Regulates Their Haematopoiesis-Supporting Ability. EBioMedicine (2020) 53:102677. doi: 10.1016/j.ebiom.2020.102677 32114389 PMC7047195

[45] Lyu ZS Tang SQ Xing T Zhou Y Lv M Fu HX. Glycolytic Enzyme Pfkfb3 Determines Bone Marrow Endothelial Progenitor Cell Damage Post Chemotherapy and Irradiation. Haematologica (2022). doi: 10.3324/haematol.2021.279756 PMC9521251 35354250

[46] Crane GM Jeffery E Morrison SJ. Adult Haematopoietic Stem Cell Niches. Nat Rev Immunol (2017) 17(9):573–90. doi: 10.1038/nri.2017.53 28604734

[47] Plein A Fantin A Denti L Pollard JW Ruhrberg C. Erythro-Myeloid Progenitors Contribute Endothelial Cells to Blood Vessels. Nature (2018) 562(7726):223–8. doi: 10.1038/s41586-018-0552-x PMC6289247 30258231

[48] Shi MM Kong Y Song Y Sun YQ Wang Y Zhang XH. Atorvastatin Enhances Endothelial Cell Function in Posttransplant Poor Graft Function. Blood (2016) 128(25):2988–99. doi: 10.1182/blood-2016-03-702803 27769957

[49] Kong Y Wang Y Zhang YY Shi MM Mo XD Sun YQ. Prophylactic Oral Nac Reduced Poor Hematopoietic Reconstitution by Improving Endothelial Cells After Haploidentical Transplantation. Blood Adv (2019) 3(8):1303–17. doi: 10.1182/bloodadvances.2018029454 PMC6482364 31015207

[50] Mesnieres M Bohm AM Peredo N Trompet D Valle-Tenney R Bajaj M. Fetal Hematopoietic Stem Cell Homing Is Controlled by Vegf Regulating the Integrity and Oxidative Status of the Stromal-Vascular Bone Marrow Niches. Cell Rep (2021) 36(8):109618. doi: 10.1016/j.celrep.2021.109618 34433017 PMC8411121

[51] Hooper AT Butler JM Nolan DJ Kranz A Iida K Kobayashi M. Engraftment and Reconstitution of Hematopoiesis Is Dependent on Vegfr2-Mediated Regeneration of Sinusoidal Endothelial Cells. Cell Stem Cell (2009) 4(3):263–74. doi: 10.1016/j.stem.2009.01.006 PMC3228275 19265665

[52] Chen Q Liu Y Jeong HW Stehling M Dinh VV Zhou B. Apelin(+) Endothelial Niche Cells Control Hematopoiesis and Mediate Vascular Regeneration After Myeloablative Injury. Cell Stem Cell (2019) 25(6):768–83.e6. doi: 10.1016/j.stem.2019.10.006 31761723 PMC6900750

[53] Zeng L Chen C Song G Yan Z Xu S Jia L. Infusion of Endothelial Progenitor Cells Accelerates Hematopoietic and Immune Reconstitution, and Ameliorates the Graft-Versus-Host Disease After Hematopoietic Stem Cell Transplantation. Cell Biochem Biophys (2012) 64(3):213–22. doi: 10.1007/s12013-012-9387-5 22806343

[54] Salter AB Meadows SK Muramoto GG Himburg H Doan P Daher P. Endothelial Progenitor Cell Infusion Induces Hematopoietic Stem Cell Reconstitution *in Vivo* . Blood (2009) 113(9):2104–7. doi: 10.1182/blood-2008-06-162941 PMC2651019 19141867

[55] Hu L Cheng H Gao Y Shi M Liu Y Hu Z. Antioxidant N-Acetyl-L-Cysteine Increases Engraftment of Human Hematopoietic Stem Cells in Immune-Deficient Mice. Blood (2014) 124(20):e45–8. doi: 10.1182/blood-2014-03-559369 PMC4231425 25287706

[56] Crippa S Santi L Berti M De Ponti G Bernardo ME. Role of *Ex Vivo* Expanded Mesenchymal Stromal Cells in Determining Hematopoietic Stem Cell Transplantation Outcome. Front Cell Dev Biol (2021) 9:663316. doi: 10.3389/fcell.2021.663316 34017834 PMC8129582

[57] Song Y Zhao HY Lyu ZS Cao XN Shi MM Wen Q. Dysfunctional Bone Marrow Mesenchymal Stem Cells in Patients With Poor Graft Function After Allogeneic Hematopoietic Stem Cell Transplantation. Biol Blood Marrow Transplant (2018) 24(10):1981–9. doi: 10.1016/j.bbmt.2018.06.021 29933074

[58] Carrancio S Romo C Ramos T Lopez-Holgado N Muntion S Prins HJ. Effects of Msc Coadministration and Route of Delivery on Cord Blood Hematopoietic Stem Cell Engraftment. Cell Transplant (2013) 22(7):1171–83. doi: 10.3727/096368912X657431 23031585

[59] Li T Luo C Zhang J Wei L Sun W Xie Q. Efficacy and Safety of Mesenchymal Stem Cells Co-Infusion in Allogeneic Hematopoietic Stem Cell Transplantation: A Systematic Review and Meta-Analysis. Stem Cell Res Ther (2021) 12(1):246. doi: 10.1186/s13287-021-02304-x 33879242 PMC8056684

[60] Liu X Wu M Peng Y Chen X Sun J Huang F. Improvement in Poor Graft Function After Allogeneic Hematopoietic Stem Cell Transplantation Upon Administration of Mesenchymal Stem Cells From Third-Party Donors: A Pilot Prospective Study. Cell Transplant (2014) 23(9):1087–98. doi: 10.3727/096368912X661319 23294601

[61] Ball LM Bernardo ME Roelofs H Lankester A Cometa A Egeler RM. Cotransplantation of *Ex Vivo* Expanded Mesenchymal Stem Cells Accelerates Lymphocyte Recovery and May Reduce the Risk of Graft Failure in Haploidentical Hematopoietic Stem-Cell Transplantation. Blood (2007) 110(7):2764–7. doi: 10.1182/blood-2007-04-087056 17638847

[62] Li R Tu J Zhao J Pan H Fang L Shi J. Mesenchymal Stromal Cells as Prophylaxis for Graft-Versus-Host Disease in Haplo-Identical Hematopoietic Stem Cell Transplantation Recipients With Severe Aplastic Anemia?-A Systematic Review and Meta-Analysis. Stem Cell Res Ther (2021) 12(1):106. doi: 10.1186/s13287-021-02170-7 33541414 PMC7860635

[63] Wang YT Kong Y Song Y Han W Zhang YY Zhang XH. Increased Type 1 Immune Response in the Bone Marrow Immune Microenvironment of Patients With Poor Graft Function After Allogeneic Hematopoietic Stem Cell Transplantation. Biol Blood Marrow Transplant (2016) 22(8):1376–82. doi: 10.1016/j.bbmt.2016.04.016 27131864

[64] Kong Y Wang YT Cao XN Song Y Chen YH Sun YQ. Aberrant T Cell Responses in the Bone Marrow Microenvironment of Patients With Poor Graft Function After Allogeneic Hematopoietic Stem Cell Transplantation. J Transl Med (2017) 15(1):57. doi: 10.1186/s12967-017-1159-y 28292332 PMC5351211

[65] Luo Y Shao L Chang J Feng W Liu YL Cottler-Fox MH. M1 and M2 Macrophages Differentially Regulate Hematopoietic Stem Cell Self-Renewal and *Ex Vivo* Expansion. Blood Adv (2018) 2(8):859–70. doi: 10.1182/bloodadvances.2018015685 PMC5916005 29666049

[66] Zhao HY Lyu ZS Duan CW Song Y Han TT Mo XD. An Unbalanced Monocyte Macrophage Polarization in the Bone Marrow Microenvironment of Patients With Poor Graft Function After Allogeneic Haematopoietic Stem Cell Transplantation. Br J Haematol (2018) 182(5):679–92. doi: 10.1111/bjh.15452 29974948

[67] Zhao HY Zhang YY Xing T Tang SQ Wen Q Lyu ZS. M2 Macrophages, But Not M1 Macrophages, Support Megakaryopoiesis by Upregulating Pi3k-Akt Pathway Activity. Signal Transduct Target Ther (2021) 6(1):234. doi: 10.1038/s41392-021-00627-y 34140465 PMC8211642

[68] Peffault de Latour R Kulasekararaj A Iacobelli S Terwel SR Cook R Griffin M. Eltrombopag Added to Immunosuppression in Severe Aplastic Anemia. N Engl J Med (2022) 386(1):11–23. doi: 10.1056/NEJMoa2109965 34986284

[69] Tang C Chen F Kong D Ma Q Dai H Yin J. Successful Treatment of Secondary Poor Graft Function Post Allogeneic Hematopoietic Stem Cell Transplantation With Eltrombopag. J Hematol Oncol (2018) 11(1):103. doi: 10.1186/s13045-018-0649-6 30115080 PMC6097332

[70] Hosokawa K Yamazaki H Tanabe M Imi T Sugimori N Nakao S. High-Dose Romiplostim Accelerates Hematologic Recovery in Patients With Aplastic Anemia Refractory to Eltrombopag. Leukemia (2021) 35(3):906–9. doi: 10.1038/s41375-020-0950-6 32616921

[71] Al-Samkari H Jiang D Gernsheimer T Liebman H Lee S Wojdyla M. Adults With Immune Thrombocytopenia Who Switched to Avatrombopag Following Prior Treatment With Eltrombopag or Romiplostim: A Multicentre Us Study. Br J Haematol (2022) 197(3):359–66. doi: 10.1111/bjh.18081 PMC9306832 35179784

[72] Ruggeri A Rocha V Masson E Labopin M Cunha R Absi L. Impact of Donor-Specific Anti-Hla Antibodies on Graft Failure and Survival After Reduced Intensity Conditioning-Unrelated Cord Blood Transplantation: A Eurocord, Societe Francophone D'histocompatibilite Et D'immunogenetique (Sfhi) and Societe Francaise De Greffe De Moelle Et De Therapie Cellulaire (Sfgm-Tc) Analysis. Haematologica (2013) 98(7):1154–60. doi: 10.3324/haematol.2012.077685 PMC3696621 23242594

[73] Ciurea SO Thall PF Wang X Wang SA Hu Y Cano P. Donor-Specific Anti-Hla Abs and Graft Failure in Matched Unrelated Donor Hematopoietic Stem Cell Transplantation. Blood (2011) 118(22):5957–64. doi: 10.1182/blood-2011-06-362111 PMC3761379 21967975

[74] Zhao X Zhao X Huo M Fan Q Pei X Wang Y. Donor-Specific Anti-Human Leukocyte Antigen Antibodies Predict Prolonged Isolated Thrombocytopenia and Inferior Outcomes of Haploidentical Hematopoietic Stem Cell Transplantation. J Immunol Res (2017) 2017:1043836. doi: 10.1155/2017/1043836 28484721 PMC5412255

[75] Fuji S Oshima K Ohashi K Sawa M Saito T Eto T. Impact of Pretransplant Donor-Specific Anti-Hla Antibodies on Cord Blood Transplantation on Behalf of the Transplant Complications Working Group of Japan Society for Hematopoietic Cell Transplantation. Bone Marrow Transplant (2020) 55(4):722–8. doi: 10.1038/s41409-019-0712-0 31591450

[76] Ciurea SO Thall PF Milton DR Barnes TH Kongtim P Carmazzi Y. Complement-Binding Donor-Specific Anti-Hla Antibodies and Risk of Primary Graft Failure in Hematopoietic Stem Cell Transplantation. Biol Blood Marrow Transplant (2015) 21(8):1392–8. doi: 10.1016/j.bbmt.2015.05.001 PMC4506716 25985919

[77] Giammarco S Raiola AM Di Grazia C Bregante S Gualandi F Varaldo R. Second Haploidentical Stem Cell Transplantation for Primary Graft Failure. Bone Marrow Transplant (2021) 56(6):1291–6. doi: 10.1038/s41409-020-01183-9 33328569

[78] Sun YQ Wang Y Wang FR Yan CH Cheng YF Chen YH. Graft Failure in Patients With Hematological Malignancies: A Successful Salvage With a Second Transplantation From a Different Haploidentical Donor. Front Med (Lausanne) (2021) 8:604085. doi: 10.3389/fmed.2021.604085 34150785 PMC8212968

[79] Srour SA Kongtim P Rondon G Chen J Petropoulos D Ramdial J. Haploidentical Transplants for Patients With Relapse After the First Allograft. Am J Hematol (2020). doi: 10.1002/ajh.25924 32619033

[80] Ciurea SO Cao K Fernandez-Vina M Kongtim P Malki MA Fuchs E. Correction: The European Society for Blood and Marrow Transplantation (Ebmt) Consensus Guidelines for the Detection and Treatment of Donor-Specific Anti-Hla Antibodies (Dsa) in Haploidentical Hematopoietic Cell Transplantation. Bone Marrow Transplant (2019) 54(5):784. doi: 10.1038/s41409-018-0332-0 30232413

[81] Chang YJ Xu LP Wang Y Zhang XH Chen H Chen YH. Rituximab for Desensitization During Hla-Mismatched Stem Cell Transplantation in Patients With a Positive Donor-Specific Anti-Hla Antibody. Bone Marrow Transplant (2020) 55(7):1326–36. doi: 10.1038/s41409-020-0928-z 32385341

[82] Yoshihara S Maruya E Taniguchi K Kaida K Kato R Inoue T. Risk and Prevention of Graft Failure in Patients With Preexisting Donor-Specific Hla Antibodies Undergoing Unmanipulated Haploidentical Sct. Bone Marrow Transplant (2012) 47(4):508–15. doi: 10.1038/bmt.2011.131 21691261

[83] Choe H Gergis U Hsu J Phillips A Shore T Christos P. Bortezomib and Immune Globulin Have Limited Effects on Donor-Specific Hla Antibodies in Haploidentical Cord Blood Stem Cell Transplantation: Detrimental Effect of Persistent Haploidentical Donor-Specific Hla Antibodies. Biol Blood Marrow Transplant (2019) 25(2):e60–e4. doi: 10.1016/j.bbmt.2018.10.018 30661542

[84] Hassan S West KA Ward WW Kanakry JA Flegel WA. Rebound and Overshoot of Donor-Specific Antibodies to Human Leukocyte Antigens (Hla) During Desensitization With Plasma Exchanges in Hematopoietic Progenitor Cell Transplantation: A Case Report. Transfusion (2021) 61(6):1980–6. doi: 10.1111/trf.16411 PMC9097367 33899963

[85] Lorant T Bengtsson M Eich T Eriksson BM Winstedt L Jarnum S. Safety, Immunogenicity, Pharmacokinetics, and Efficacy of Degradation of Anti-Hla Antibodies by Ides (Imlifidase) in Chronic Kidney Disease Patients. Am J Transplant (2018) 18(11):2752–62. doi: 10.1111/ajt.14733 PMC6221156 29561066

[86] Allhorn M Briceno JG Baudino L Lood C Olsson ML Izui S. The Igg-Specific Endoglycosidase Endos Inhibits Both Cellular and Complement-Mediated Autoimmune Hemolysis. Blood (2010) 115(24):5080–8. doi: 10.1182/blood-2009-08-239020 PMC2890147 20357243

[87] Lin J Boon L Bockermann R Robertson AK Kjellman C Anderson CC. Desensitization Using Imlifidase and Endos Enables Chimerism Induction in Allosensitized Recipient Mice. Am J Transplant (2020) 20(9):2356–65. doi: 10.1111/ajt.15851 PMC7496317 32185855

[88] Xiao Y Song J Jiang Z Li Y Gao Y Xu W. Risk-Factor Analysis of Poor Graft Function After Allogeneic Hematopoietic Stem Cell Transplantation. Int J Med Sci (2014) 11(6):652–7. doi: 10.7150/ijms.6337 PMC4021098 24834012

[89] Zhou JR Shi DY Wei R Wang Y Yan CH Zhang XH. Co-Reactivation of Cytomegalovirus and Epstein-Barr Virus Was Associated With Poor Prognosis After Allogeneic Stem Cell Transplantation. Front Immunol (2020) 11:620891. doi: 10.3389/fimmu.2020.620891 33664733 PMC7921792

[90] Reddehase MJ Holtappels R Lemmermann NAW. Consequence of Histoincompatibility Beyond Gvh-Reaction in Cytomegalovirus Disease Associated With Allogeneic Hematopoietic Cell Transplantation: Change of Paradigm. Viruses (2021) 13(8):1530. doi: 10.3390/v13081530 34452395 PMC8402734

[91] Degli-Esposti MA Hill GR. Immune Control of Cytomegalovirus Reactivation in Stem Cell Transplantation. Blood (2021) 139(9):1277–88. doi: 10.1182/blood.2020010028 34166512

[92] Suessmuth Y Mukherjee R Watkins B Koura DT Finstermeier K Desmarais C. Cmv Reactivation Drives Posttransplant T-Cell Reconstitution and Results in Defects in the Underlying Tcrbeta Repertoire. Blood (2015) 125(25):3835–50. doi: 10.1182/blood-2015-03-631853 PMC4473113 25852054

[93] Yeh AC Varelias A Reddy A Barone SM Olver S Chilson K. Cmv Exposure Drives Long-Term Cd57+ Cd4 Memory T Cell Inflation Following Allogeneic Stem Cell Transplant. Blood (2021) 138(26):2874–85. doi: 10.1182/blood.2020009492 PMC8718626 34115118

[94] Akahoshi Y Kimura SI Inamoto Y Seo S Muranushi H Shimizu H. Effect of Cytomegalovirus Reactivation With or Without Acute Graft-Versus-Host Disease on the Risk of Nonrelapse Mortality. Clin Infect Dis (2021) 73(3):e620–e8. doi: 10.1093/cid/ciaa1871 33341890

[95] Takenaka K Nishida T Asano-Mori Y Oshima K Ohashi K Mori T. Cytomegalovirus Reactivation After Allogeneic Hematopoietic Stem Cell Transplantation Is Associated With a Reduced Risk of Relapse in Patients With Acute Myeloid Leukemia Who Survived to Day 100 After Transplantation: The Japan Society for Hematopoietic Cell Transplantation Transplantation-Related Complication Working Group. Biol Blood Marrow Transplant (2015) 21(11):2008–16. doi: 10.1016/j.bbmt.2015.07.019 26211985

[96] Teira P Battiwalla M Ramanathan M Barrett AJ Ahn KW Chen M. Early Cytomegalovirus Reactivation Remains Associated With Increased Transplant-Related Mortality in the Current Era: A Cibmtr Analysis. Blood (2016) 127(20):2427–38. doi: 10.1182/blood-2015-11-679639 PMC4874224 26884374

[97] Turki AT Tsachakis-Muck N Leserer S Crivello P Liebregts T Betke L. Impact of Cmv Reactivation on Relapse of Acute Myeloid Leukemia After Hct Is Dependent on Disease Stage and Atg. Blood Adv (2021) 6(1):28–36. doi: 10.1182/bloodadvances.2021005509 PMC8753205 34619756

[98] Leserer S Bayraktar E Trilling M Bogdanov R Arrieta-Bolanos E Tsachakis-Muck N. Cytomegalovirus Kinetics After Hematopoietic Cell Transplantation Reveal Peak Titers With Differential Impact on Mortality, Relapse and Immune Reconstitution. Am J Hematol (2021) 96(4):436–45. doi: 10.1002/ajh.26094 33439488

[99] Einsele H Ljungman P Boeckh M. How I Treat Cmv Reactivation After Allogeneic Hematopoietic Stem Cell Transplantation. Blood (2020) 135(19):1619–29. doi: 10.1182/blood.2019000956 PMC7484743 32202631

[100] Ljungman P de la Camara R Milpied N Volin L Russell CA Crisp A. Randomized Study of Valacyclovir as Prophylaxis Against Cytomegalovirus Reactivation in Recipients of Allogeneic Bone Marrow Transplants. Blood (2002) 99(8):3050–6. doi: 10.1182/blood.v99.8.3050 11929799

[101] Selby PR Shakib S Peake SL Warner MS Yeung D Hahn U. A Systematic Review of the Clinical Pharmacokinetics, Pharmacodynamics and Toxicodynamics of Ganciclovir/Valganciclovir in Allogeneic Haematopoietic Stem Cell Transplant Patients. Clin Pharmacokinet (2021) 60(6):727–39. doi: 10.1007/s40262-020-00982-z 33515202

[102] Imlay HN Kaul DR. Letermovir and Maribavir for the Treatment and Prevention of Cytomegalovirus Infection in Solid Organ and Stem Cell Transplant Recipients. Clin Infect Dis (2021) 73(1):156–60. doi: 10.1093/cid/ciaa1713 33197929

[103] Metafuni E Chiusolo P Sica S Laurenti L Bregante S Van Lint MT. Foscarnet Treatment of Cytomegalovirus Infection in Haploidentical or Unrelated Donor Transplants. Bone Marrow Transplant (2018) 53(12):1560–7. doi: 10.1038/s41409-018-0200-y PMC6281566 29795416

[104] Marty FM Ljungman P Chemaly RF Maertens J Dadwal SS Duarte RF. Letermovir Prophylaxis for Cytomegalovirus in Hematopoietic-Cell Transplantation. N Engl J Med (2017) 377(25):2433–44. doi: 10.1056/NEJMoa1706640 29211658

[105] Mori Y Jinnouchi F Takenaka K Aoki T Kuriyama T Kadowaki M. Efficacy of Prophylactic Letermovir for Cytomegalovirus Reactivation in Hematopoietic Cell Transplantation: A Multicenter Real-World Data. Bone Marrow Transplant (2021) 56(4):853–62. doi: 10.1038/s41409-020-01082-z 33139867

[106] Sassine J Khawaja F Shigle TL Handy V Foolad F Aitken S. Refractory and Resistant Cytomegalovirus After Hematopoietic Cell Transplant in the Letermovir Primary Prophylaxis Era. Clin Infect Dis (2021) 73(8):1346–54. doi: 10.1093/cid/ciab298 PMC8528390 33830182

[107] Ljungman P Schmitt M Marty FM Maertens J Chemaly RF Kartsonis NA. A Mortality Analysis of Letermovir Prophylaxis for Cytomegalovirus (Cmv) in Cmv-Seropositive Recipients of Allogeneic Hematopoietic Cell Transplantation. Clin Infect Dis (2020) 70(8):1525–33. doi: 10.1093/cid/ciz490 PMC7146004 31179485

[108] Zamora D Duke ER Xie H Edmison BC Akoto B Kiener R. Cytomegalovirus-Specific T-Cell Reconstitution Following Letermovir Prophylaxis After Hematopoietic Cell Transplantation. Blood (2021) 138(1):34–43. doi: 10.1182/blood.2020009396 33657225 PMC8493975

[109] Kumar D Mian M Singer L Humar A. An Interventional Study Using Cell-Mediated Immunity to Personalize Therapy for Cytomegalovirus Infection After Transplantation. Am J Transplant (2017) 17(9):2468–73. doi: 10.1111/ajt.14347 28500691

[110] Adler SP Lewis N Conlon A Christiansen MP Al-Ibrahim M Rupp R. Phase 1 Clinical Trial of a Conditionally Replication-Defective Human Cytomegalovirus (Cmv) Vaccine in Cmv-Seronegative Subjects. J Infect Dis (2019) 220(3):411–9. doi: 10.1093/infdis/jiz141 31535143

[111] Maertens J Cordonnier C Jaksch P Poire X Uknis M Wu J. Maribavir for Preemptive Treatment of Cytomegalovirus Reactivation. N Engl J Med (2019) 381(12):1136–47. doi: 10.1056/NEJMoa1714656 31532960

[112] Marty FM Winston DJ Rowley SD Vance E Papanicolaou GA Mullane KM. Cmx001 to Prevent Cytomegalovirus Disease in Hematopoietic-Cell Transplantation. N Engl J Med (2013) 369(13):1227–36. doi: 10.1056/NEJMoa1303688 24066743

[113] Schleiss MR. Recombinant Cytomegalovirus Glycoprotein B Vaccine: Rethinking the Immunological Basis of Protection. Proc Natl Acad Sci USA (2018) 115(24):6110–2. doi: 10.1073/pnas.1806420115 PMC6004476 29875141

[114] De Groof TWM Elder EG Lim EY Heukers R Bergkamp ND Groves IJ. Targeting the Latent Human Cytomegalovirus Reservoir for T-Cell-Mediated Killing With Virus-Specific Nanobodies. Nat Commun (2021) 12(1):4436. doi: 10.1038/s41467-021-24608-5 34290252 PMC8295288

[115] Menger L Gouble A Marzolini MA Pachnio A Bergerhoff K Henry JY. Talen-Mediated Genetic Inactivation of the Glucocorticoid Receptor in Cytomegalovirus-Specific T Cells. Blood (2015) 126(26):2781–9. doi: 10.1182/blood-2015-08-664755 26508783

[116] Malki MMA Song JY Yang D Cao T Aldoss I Mokhtari S. Iron Overload Is Associated With Delayed Engraftment and Increased Nonrelapse Mortality in Recipients of Umbilical Cord Blood Hematopoietic Cell Transplantation. Biol Blood Marrow Transplant (2020) 26(9):1697–703. doi: 10.1016/j.bbmt.2020.06.002 PMC7486229 32534103

[117] Penack O Peczynski C van der Werf S Finke J Ganser A Schoemans H. Association of Serum Ferritin Levels Before Start of Conditioning With Mortality After Allosct - a Prospective, Non-Interventional Study of the Ebmt Transplant Complications Working Party. Front Immunol (2020) 11:586. doi: 10.3389/fimmu.2020.00586 32351502 PMC7174614

[118] Trottier BJ Burns LJ DeFor TE Cooley S Majhail NS. Association of Iron Overload With Allogeneic Hematopoietic Cell Transplantation Outcomes: A Prospective Cohort Study Using R2-Mri-Measured Liver Iron Content. Blood (2013) 122(9):1678–84. doi: 10.1182/blood-2013-04-499772 PMC3953015 23777771

[119] Dallalio G Law E Means RT Jr. Hepcidin Inhibits *in Vitro* Erythroid Colony Formation at Reduced Erythropoietin Concentrations. Blood (2006) 107(7):2702–4. doi: 10.1182/blood-2005-07-2854 PMC1895381 16332970

[120] Sakamoto S Kawabata H Kanda J Uchiyama T Mizumoto C Kitano T. High Pretransplant Hepcidin Levels Are Associated With Poor Overall Survival and Delayed Platelet Engraftment After Allogeneic Hematopoietic Stem Cell Transplantation. Cancer Med (2017) 6(1):120–8. doi: 10.1002/cam4.974 PMC5269567 27905193

[121] Iolascon A Andolfo I Russo R. Congenital Dyserythropoietic Anemias. Blood (2020) 136(11):1274–83. doi: 10.1182/blood.2019000948 32702750

[122] Kautz L Nemeth E. Molecular Liaisons Between Erythropoiesis and Iron Metabolism. Blood (2014) 124(4):479–82. doi: 10.1182/blood-2014-05-516252 PMC4110655 24876565

[123] Jin X He X Cao X Xu P Xing Y Sui S. Iron Overload Impairs Normal Hematopoietic Stem and Progenitor Cells Through Reactive Oxygen Species and Shortens Survival in Myelodysplastic Syndrome Mice. Haematologica (2018) 103(10):1627–34. doi: 10.3324/haematol.2018.193128 PMC6165791 29903757

[124] Zheng Q Zhao Y Guo J Zhao S Fei C Xiao C. Iron Overload Promotes Mitochondrial Fragmentation in Mesenchymal Stromal Cells From Myelodysplastic Syndrome Patients Through Activation of the Ampk/Mff/Drp1 Pathway. Cell Death Dis (2018) 9(5):515. doi: 10.1038/s41419-018-0552-7 29725013 PMC5938711

[125] Okabe H Suzuki T Uehara E Ueda M Nagai T Ozawa K. The Bone Marrow Hematopoietic Microenvironment Is Impaired in Iron-Overloaded Mice. Eur J Haematol (2014) 93(2):118–28. doi: 10.1111/ejh.12309 24628561

[126] Tenneti P Chojecki A Knovich MA. Iron Overload in the Hct Patient: A Review. Bone Marrow Transplant (2021) 56(8):1794–804. doi: 10.1038/s41409-021-01244-7 33782548

[127] Vlachodimitropoulou E Chen YL Garbowski M Koonyosying P Psaila B Sola-Visner M. Eltrombopag: A Powerful Chelator of Cellular or Extracellular Iron(Iii) Alone or Combined With a Second Chelator. Blood (2017) 130(17):1923–33. doi: 10.1182/blood-2016-10-740241 PMC6558288 28864815

[128] Kao YR Chen J Narayanagari SR Todorova TI Aivalioti MM Ferreira M. Thrombopoietin Receptor-Independent Stimulation of Hematopoietic Stem Cells by Eltrombopag. Sci Transl Med (2018) 10(458):eaas9536. doi: 10.1126/scitranslmed.aas9563 PMC9899005 30209246

[129] Gardenghi S Ramos P Marongiu MF Melchiori L Breda L Guy E. Hepcidin as a Therapeutic Tool to Limit Iron Overload and Improve Anemia in Beta-Thalassemic Mice. J Clin Invest (2010) 120(12):4466–77. doi: 10.1172/JCI41717 PMC2993583 21099112

[130] Li H Rybicki AC Suzuka SM von Bonsdorff L Breuer W Hall CB. Transferrin Therapy Ameliorates Disease in Beta-Thalassemic Mice. Nat Med (2010) 16(2):177–82. doi: 10.1038/nm.2073 20098432

